# Validation testing of five home blood pressure monitoring devices for the upper arm according to the ISO 81060-2:2018/AMD 1:2020 protocol

**DOI:** 10.1038/s41371-022-00795-6

**Published:** 2023-01-18

**Authors:** Yaw Amofa Peprah, Ji Young Lee, Stephen D. Persell

**Affiliations:** 1 Division of General Internal Medicine, Department of Medicine, Feinberg School of Medicine, Northwestern University, Chicago, IL USA; 2 Center for Primary Care Innovation, Institute for Public Health and Medicine, Feinberg School of Medicine, Northwestern University, Chicago, IL USA

**Keywords:** Hypertension, Diagnosis, Heart failure

## Abstract

The accuracy of Omron 10 Series BP7450 (HEM-7342T-Z), Omron Platinum BP5450 (HEM-7343T-Z), Walmart Equate Premium 8000 Series UA-8000WM, Walgreens Premium 15+ WGNBPA-960BT, and CVS Series 800 BP3MW1-4YCVS were assessed in an adult general population compared to a mercury sphygmomanometer standard according to the ISO 81060-2:2018/AMD 1:2020 validation procedure. Omron selected the monitors and included three non-Omron monitors because they were from large retail vendors in the United States and these monitors did not have accessible results from validation testing. The BP7450, *N* = 85, passed both criteria for the standard. Mean (SD) differences in paired SBP and DBP determinations between the test device and reference were 0.5 (7.7) and 2.5 (6.8) mm Hg. The BP5450, *N* = 86, passed both criteria. Mean (SD) differences in paired SBP and DBP determinations were 1.9 (7.0) and 3.6 (6.4) mm Hg. The UA-8000WM, *N* = 85, did not meet the first criterion for the standard. Mean (SD) differences in paired SBP and DBP determinations were 2.5 (8.0) and 5.1 (6.4) mm Hg. The WGNBPA-240BT, *N* = 85, did not meet the first criterion for the standard. Mean (SD) differences in paired SBP and DBP determinations were 7.9 (8.5) and 5.5 (6.7) mm Hg. The BP3MW1-4YCVS, *N* = 85, did not meet the first criterion for the standard. Mean (SD) differences in paired SBP and DBP determinations were 5.8 (8.7) and 3.1 (5.6) mm Hg. These findings emphasize the importance of verifying the validation status of home blood pressure monitors before use by consumers.

## Introduction

Out-of-office blood pressure measurement is widely recommended due to its ability to provide more accurate measurements to guide diagnosis and treatment compared to infrequent office BP measurements. Multiple guidelines recommend out-of-office blood pressure measurement for hypertension diagnosis and management [1–4]. Accurate blood pressure measurement is important when diagnosing and treating hypertension [5], since inaccuracy in blood pressure measurement values can result in over diagnosis or under diagnosis as well as overtreatment or under treatment of hypertension [6]. However, many marketed blood pressure monitors currently have not been validated and are inaccurate [7]. As a result, consumers may unknowingly end up with an inaccurate BP monitor purchased from the market. Moreover, many consumers may be unaware of the existence of a website that helps them determine the validation status of available monitors.

Retailers provide blood pressure monitors for home use without access to published results of validation testing. The aim of this study was to determine the accuracy of five digital blood pressure monitors intended for home use that are widely available through major retailers in the US. Omron 10 Series BP7450 (HEM-7342T-Z made by Omron Healthcare Co., Ltd., Kyoto, Japan), Omron Platinum BP5450 (HEM-7343T-Z made by Omron Healthcare Co., Ltd., Kyoto, Japan), Walmart Equate Premium 8000 Series UA-8000WM (Manufacturer part number wmtbpa-240bt, distributed by HoMedics, LLC, Commerce Township, MI. USA), Walgreens Premium 15+ WGNBPA-960BT (made and distributed exclusively for Walgreens by HoMedics, LLC, Commerce Township, MI. USA), and CVS Series 800 BP3MW1-4YCVS (manufactured by Microlife USA Inc, Clearwater, FL. USA and distributed by CVS Pharmacy, Inc) in a general adult population using the validation requirements of the American National Standards Institute/Association for the Advancement of Medical Instrumentation/International Organization for Standardization (ISO 81060-2:2018/AMD 1:2020) standard [8, 9]. Omron Healthcare, Inc. contracted with investigators at Northwestern University to conduct an independent validation study of two Omron monitors and three additional monitors under the same conditions. Omron selected the non-Omron devices to be included in the study because these vendors represent a major part of the market for consumers to purchase blood pressure monitors across the United States, and these retail-available monitors did not have accessible published results of validation testing. This study is important because it tests the validity of 5 monitors sold by 4 of the large retail brands on the U.S. market under the same test conditions.

## Methods

### Devices

All of the 5 devices used in the study are automatic oscillometric devices for BP measurements on the upper arm. BP7450 has the preformed cuff called the ComFit Cuff, covering arm circumference range of 22–42 cm (9–17 inches); and the BP5450 has wide range D-ring cuff covering arm circumference range of 22–42 cm (9–17 inches) and a small D-Ring cuff for adult arm circumferences of 17–22 cm (7–9 inches). UA-8000WM is has a D-Ring cuff that covers arm circumference range of 23–43 cm (9–17 inches). WGNBPA-240BT has an UltraSoft™ D-Ring cuff that covers arm circumference range of 23–43 cm (9–17 inches). BP3MW1-4YCVS has a SoftFit™ D-Ring cuff that covers arm circumference range of 22–42 cm (9–17 inches). Two units of study devices were provided by study sponsor with a written statement that BP7450 and BP5450 were obtained from the sponsors’ inventory at their warehouse, whereas UA-8000WM, WGNBPA-240BT, and BP3MW1-4YCVS monitors were purchased directly from their corresponding retail stores. These three devices were selected because they are major retail store branded blood pressure monitors with more functions and features than some other store branded monitors. Therefore, consumers might view such monitors as being of above average quality. One of each monitor was randomly selected out of the 2 units and used for the entire duration of the study.

### Participants

Study participants who were 18 years and older with arm circumference of 17 cm to 43 cm were recruited from the general public using IRB approved research study flyers. Staff and patients of Northwestern University and Northwestern Medicine could also participate. We also recruited from patients identified through searches of the Northwestern Medicine Electronic Data Warehouse to identify potential participants with low body mass index who would be more likely to have a small arm circumference and to identify individuals with elevated blood pressures. We screened participants who met the age, arm circumference and BP readings criteria and consented them into the study. Participants who did not meet the study inclusion criteria were excluded from participating in the study.

### Study team

The study was conducted by an experienced project manager (study supervisor) and 2 observers who were all trained and certified in the basic Shared Care Research and Education, Inc. Stateline Nevada, USA blood pressure measurement training program using the American Heart Association (AHA) guidelines, with additional training on how to conduct an Association for the Advancement Medical Instrumentation (AAMI) protocol for validations of automatic BP devices.

### Blood pressure measurements

Northwestern University’s Institutional Review Board reviewed and approved the study. After obtaining written informed consent forms, participants’ arms were measured as a reference to select a corresponding cuff size for BP measurements. Participants rested for 5 minutes before baseline BP readings were taken with the reference sphygmomanometer. Two observers listened through a connected y-tube standard mercury sphygmomanometers that was calibrated before the study began. Using a dual head teaching stethoscope, the observers recorded simultaneous reference auscultatory BP measurements blinded to the other observer’s reading.

The independent readings were handed to a third study staff member, a study supervisor who determined whether the readings were within 4 mm Hg. When the two observers’ readings differed by more than 4 mm Hg, those readings were discarded in accordance with the ISO 81060-2:2018/AMD 1:2020 validation procedure. Both observers were blinded to each other’s readings and to the results of the device under test. The test device was also used to obtain an initial reading. After both baseline readings were recorded, four reference mercury sphygmomanometer measurements were taken alternately with three test device measurements with 60 second rests observed between readings. Additional readings were taken to replace the excluded readings in order to complete three valid sets of reference, test device, reference BP comparisons. Readings were excluded for pre-specified observer differences, blood pressure reading variation, participant body movement, talking or when one or both observers were unable to hear a reading. The subject was excluded if readings were repeated for a third time and the two observer measurements were not within 4 mmHg. Furthermore, subjects with BP variability in reference BP measurements >12/8 mm Hg between any two of the four reference BP measurements were excluded with the option to include from these subjects in the analysis two (instead of the three) consecutive valid pairs of reference measurements that fulfill the requirement provided this applied to no more than 10% of subjects.

### Identification of the final study cohorts

We aimed to recruit participants who had an adequate distribution of SBP, DBP, sex, and arm circumference to satisfy the requirements of the ISO 81060-2:2018/AMD 1:2020 standard. In order to reduce the burden of recruiting participants with less common characteristics (e.g., very small arm circumference, higher diastolic blood pressure) in some cases we recruited more than the 85 required participants who had eligible measurements. We used a blinded backwards removal process based solely on the participants’ initial mercury sphygmomanometer readings, sex and arm circumference—whoever was most recently recruited was examined and if they were not needed to fulfill the distribution requirements of arm circumference, SBP, DBP or sex they were removed not to be in excess of 85 subjects.

### Statistical analysis

We assessed means and standard deviations of the absolute values of the differences between the SBP and DBP measurements of the devices under test and the reference standard and compared these to Criterion 1 of the ISO 81060-2:2018/AMD 1:2020 standard, namely whether or not the means were ≤5.0 mm Hg and the standard deviation was ≤8.0 mm Hg. We made Bland-Altman plots of the differences in SBP and DBP determinations by blood pressure (means of test device and observer measurements) and arm circumference values. We calculated the standard deviations of the differences of the averaged paired determinations of the device under test and the reference measurement for each subject and compared these to Criterion 2 of the ISO 81060-2:2018/AMD 1:2020 standard.

## Results

The number of participants screened, number ineligible, the number removed by blinded backwards removal and the number included in the analytic cohorts are detailed in Table 1. The blinded backward removal process successfully identified cohorts of 85 subjects who met the distribution requirements of the ISO 81060-2:2018/AMD 1:2020 standard for SBP, DBP sex, and arm circumference for each device. After evaluation against the validation criteria, the first criterion was met for the BP5450 with *N* = 85 participants. The standard deviation of the subject-level diastolic blood pressure difference did not meet the second criterion and was within 0.1 mm Hg of the permissible difference [mean DBP difference 3.67 (5.93)]. With the addition of the next eligible subject, the distribution requirements were still met and the second criterion was also met. For the BP5450 (*N* = 86) the small cuff was tested in 14 subjects, mean SBP difference (SD) −1.5 (4.3), mean DBP difference (SD) −0.2 (5.1). The large cuff was tested in 72, mean SBP difference (SD) 2.5 (6.2), mean DBP difference (SD) 4.30 (5.8). Participant characteristics of the cohorts for all 5 devices are presented in Table 2. The test cohorts fulfilled requirements for participant arm circumference distribution in each case. Participant limb size distribution is presented in Supplementary Table 1. The test cohorts fulfilled requirements for sex, age, cuff distribution, SBP and DBP [8, 9].

**Table 1:** Participant screening and recruitment details.

	Blood Pressure Monitor Model				
	BP7450	BP5450	UA-8000WM	WGNBPA-240BT	BP3MW1-4YCVS
Total screened	88	126	107	106	110
Total excluded	3	7	2	6	3
Arrhythmia	0	0	0	0	0
Poor quality sounds	0	0	0	0	0
Any two reference SBP differ by more than 12 mm Hg or DBP by more than 8 mm Hg	1	5	2	3	2
Inter-observer difference exceeds standard	0	0	0	1	0
Persistent movement during measurement	0	0	0	0	0
Arm circumference outside of range	0	0	0	0	0
Pregnant	0	0	0	0	0
Other (participant body movement, talking, BP Variation, Upper arm Circumference <17 cm or >43 cm, etc)	2	2	0	2	1
Total removed through blinded backwards selection	0	33	20	15	22
Total included in analytic cohort	85	86	85	85	85

**Table 2:** Participant characteristics and reference blood pressure distribution.

Blood Pressure Monitor Model					
	BP7450	BP5450	UA-8000WM	WGNBPA-240BT	BP3MW1-4YCVS
Male (*N*): Female (*N*)	30: 55	26: 60	26: 59	26: 59	26: 59
Age (years)					
Range (Low: High)	18: 86	21: 87	21: 83	21: 86	21: 86
Mean (SD)	47.2 (20.3)	48.9 (20.3)	44.0 (17.8)	47.3 (19.5)	45.6 (18.8)
Arm Circumference (cm)					
Range (Low: High)	22: 42	18.5: 42	24: 42	23: 42	22: 42
Mean (SD)	31.8 (5.4)	30.7 (6.7)	32.9 (5.3)	32.8 (5.3)	32.2 (5.6)
SBP (mmHg) Overall Range (Low: High)	83.5: 207.5	80: 202.5	84: 202.5	85: 201	85: 198
≥160 (*N*: %)	16: 6.3	15: 5.8	14: 5.5	13: 5.1	14: 5.5
≥140	59: 23.1	52: 20.2	52: 20.4	51: 20.0	51: 20.0
≤100	54: 21.2	55: 21.3	62: 24.3	53: 20.8	53: 20.8
DBP (mmHg) Overall Range (Low: High)	50: 114	46.5: 113	43.5: 115.5	45.5: 111.5	45: 114.5
≥ 100 (*N*: %)	16: 6.3	13: 5.0	16: 6.3	15: 5.9	16: 6.3
≥ 85	57: 22.3	54: 20.9	51: 20.0	51: 20.0	51: 20.0
≤ 60	40: 15.7	43: 16.7	41: 16.1	46: 18.0	45: 17.6

The comparison of the test devices BP measurements and the reference measurements are provided in Table 2. Two devices had mean SBP and mean DBP differences of 5.0 mm Hg or less with SD no greater than 8.0 mm Hg (BP7450 and BP5450). One device did not meet the SBP criterion, BP3MW1-4YCVS, mean (SD) SBP difference 5.9 (8.7) mm Hg. Two devices did not meet the SBP or DBP criteria, UA-8000WM, mean (SD) SBP 2.5 (8.0) mm Hg and mean (SD) DBP 5.1 (6.4) mm Hg, and WGNBPA-240BT, mean (SD) SBP 7.9 (8.5) mm Hg and mean (SD) DBP 5.5 (6.7) mm Hg (ISO 81060-2:2018/AMD 1:2020 Criterion 1). BP7450 and BP5450 both met Criterion 2, although the SD of DBP difference at the subject level for BP5450 was borderline (Table 3).

**Table 3:** Comparison of Tested Device BP Measurements with Reference Measurements.

Device	Pairs of Measurements, *N*	SBP mean difference, mm Hg (SD)	DBP mean difference, mm Hg (SD)
**BP7450**	255	0.5 (7.7)	2.5 (6.8)
**BP5450**	255	1.9 (7.0)	3.7 (6.5)
**BP5450**	258	1.9 (7.0)	3.6 (6.4)
**UA-8000WM**	255	2.5 (8.0)^a^	5.1^a^ (6.4)
**WGNBPA-240BT**	255	7.9^a^ (8.5)^a^	5.5^a^ (6.7)
**BP3MW1-4YCVS**	255	5.9^a^ (8.7)^a^	3.1 (5.6)
**Subject Level**			
	**Subjects,** ***N***	**SD of SBP mean difference, mm Hg/Maximum permissible SD**	**SD of DBP mean difference, mm Hg/Maximum permissible SD**
**BP7450**	85	6.19/6.92	5.90/6.47
**BP5450**	85	6.10/6.68	5.93^b^/5.89
**BP5450**	86	6.08/6.68	5.89/5.89
**UA-8000WM**	85	6.19/6.47	5.40^b^/-
**WGNBPA-240BT**	85	6.64^b^/-	6.00^b^/-
**BP3MW1-4YCVS**	85	7.57^b^/-	5.02 ^b^/6.20

^a^Does not meet Criterion 1 of the ISO 81060-2:2018 + A1:2020 standard.

^b^Does not meet Criterion 2 of the ISO 81060-2:2018 + A1:2020 standard.

**Fig. 1:**
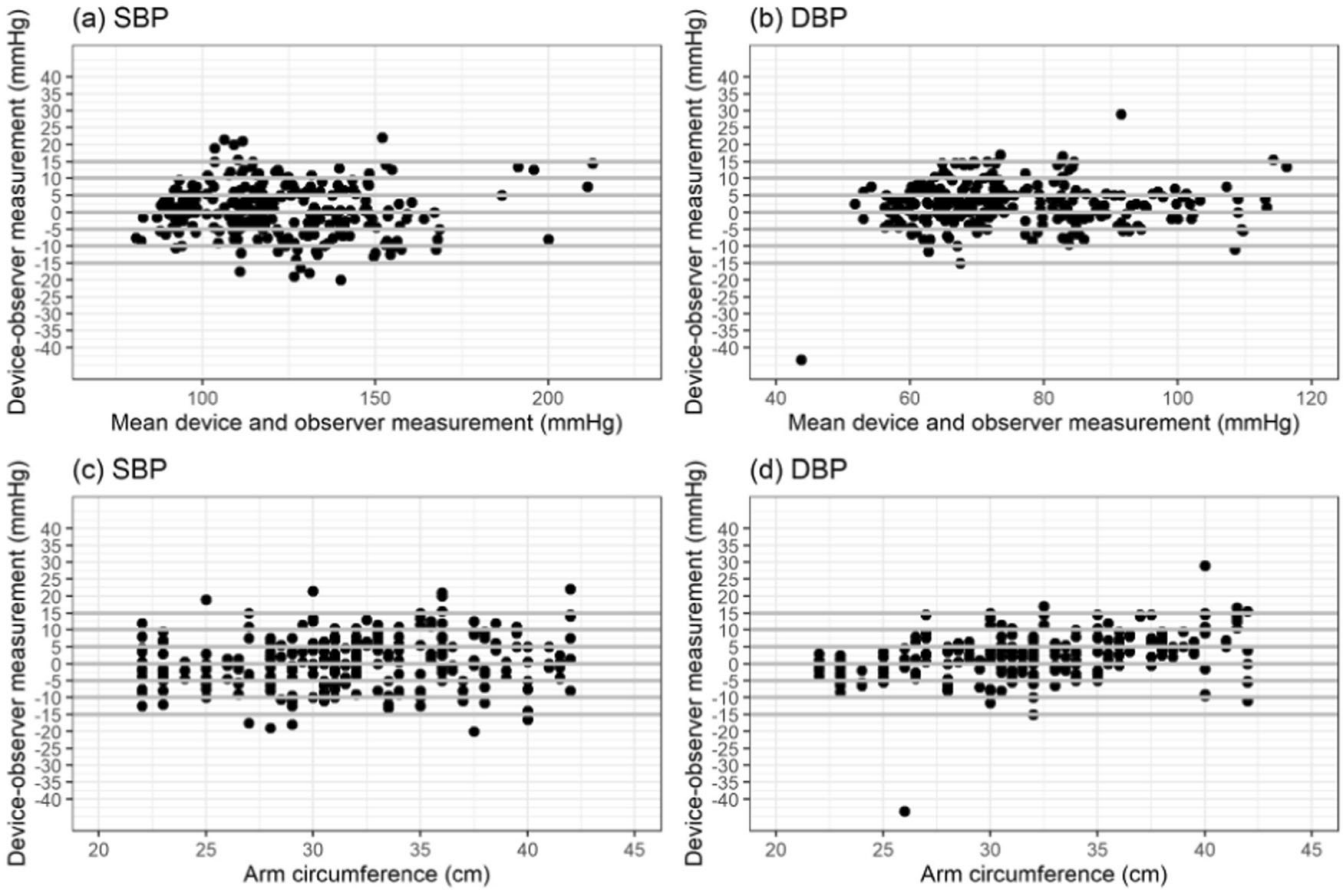
Bland-Altman Plots for BP7450. Bland–Altman Plots of the differences between the BP7450 measurements and observer measurements for (**a**) systolic blood pressure (SBP) and (**b**) diastolic blood pressure (DBP) and for (**c**) SBP by arm circumference and (**d**) DBP by arm circumference.

**Fig. 2:**
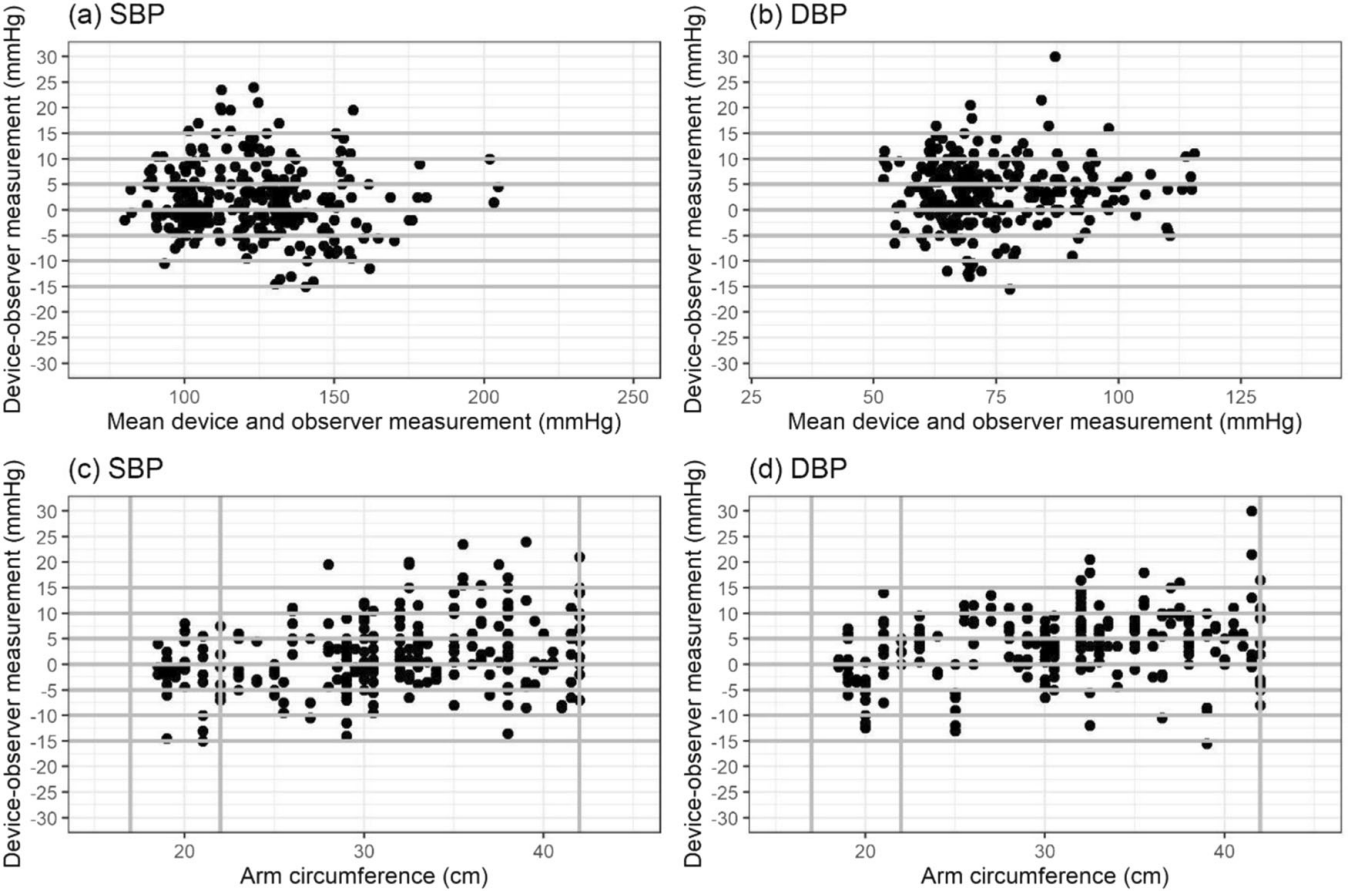
Bland-Altman Plots for BP5450. Bland–Altman Plots of the differences between the BP5450 measurements and observer measurements for (**a**) systolic blood pressure (SBP) and (**b**) diastolic blood pressure (DBP) and for (**c**) SBP by arm circumference and (**d**) DBP by arm circumference.

**Fig. 3:**
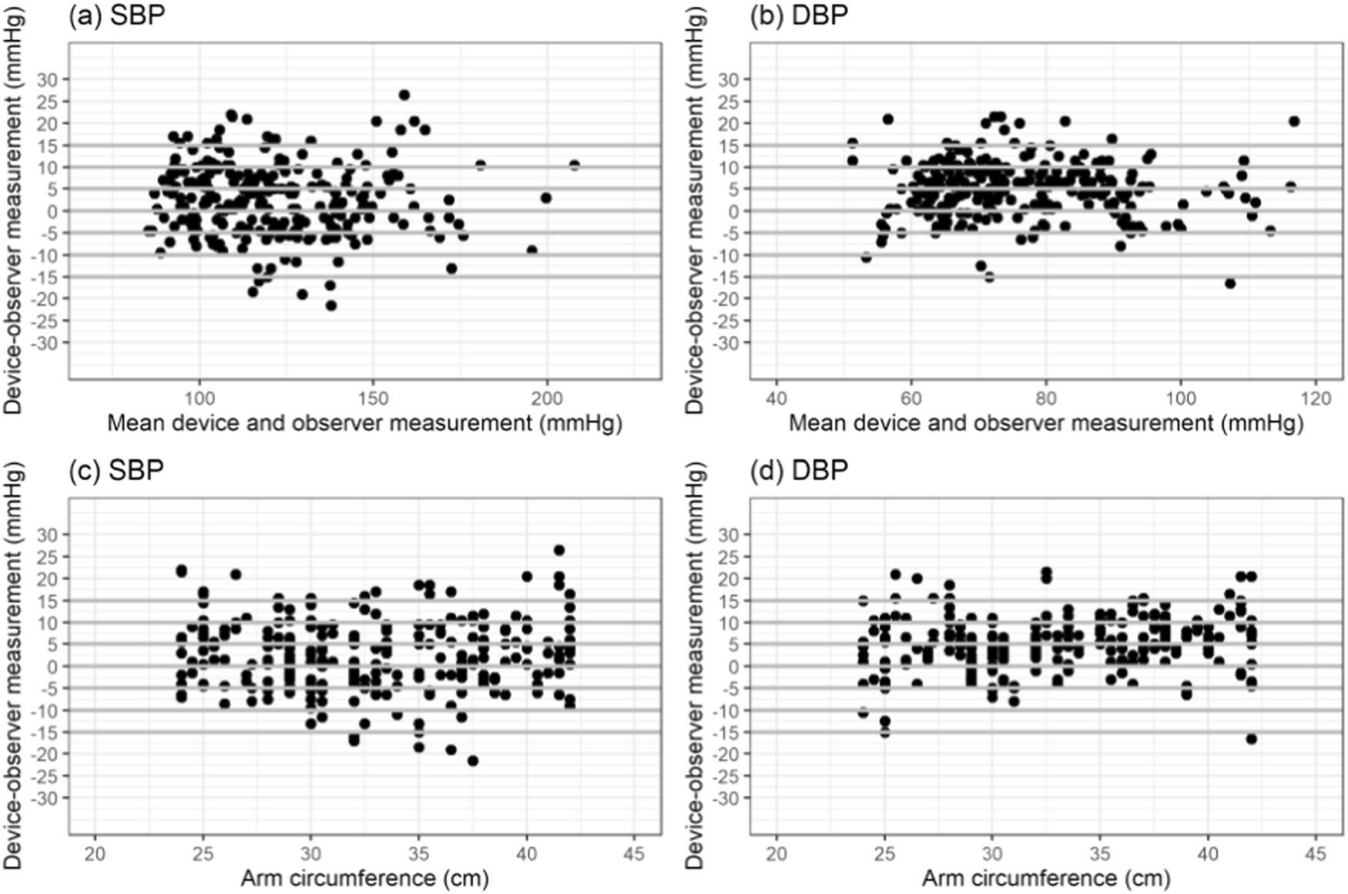
Bland-Altman Plots for UA-8000WM. Bland–Altman Plots of the differences between the UA-8000WM measurements and observer measurements for (**a**) systolic blood pressure (SBP) and (**b**) diastolic blood pressure (DBP) and for (**c**) SBP by arm circumference and (**d**) DBP by arm circumference.

**Fig. 4:**
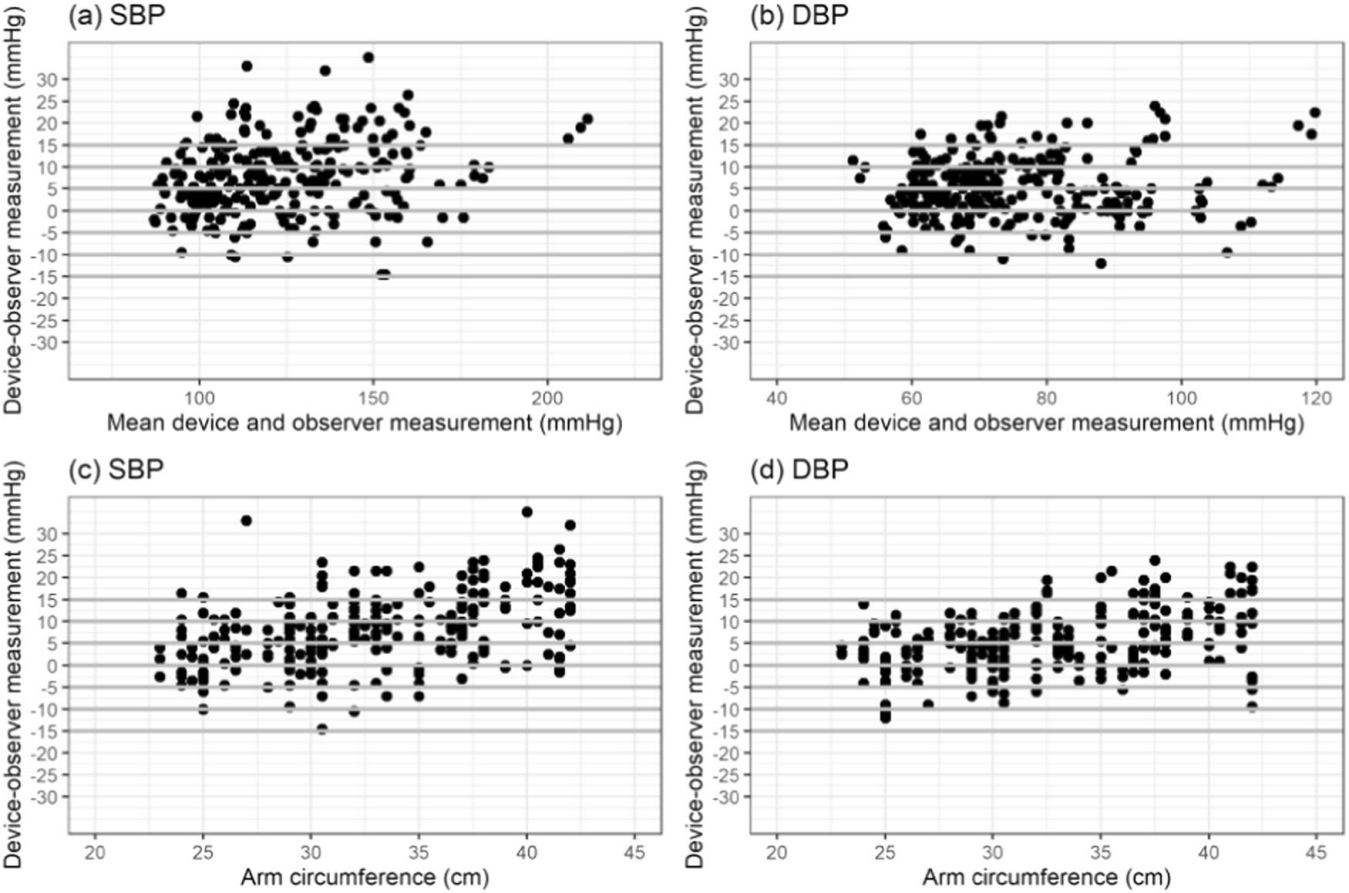
Bland-Altman Plots for WGNBPA-240BT. Bland–Altman Plots of the differences between the WGNBPA-240BT measurements and observer measurements for (**a**) systolic blood pressure (SBP) and (**b**) diastolic blood pressure (DBP) and for (**c**) SBP by arm circumference and (**d**) DBP by arm circumference.

**Fig. 5:**
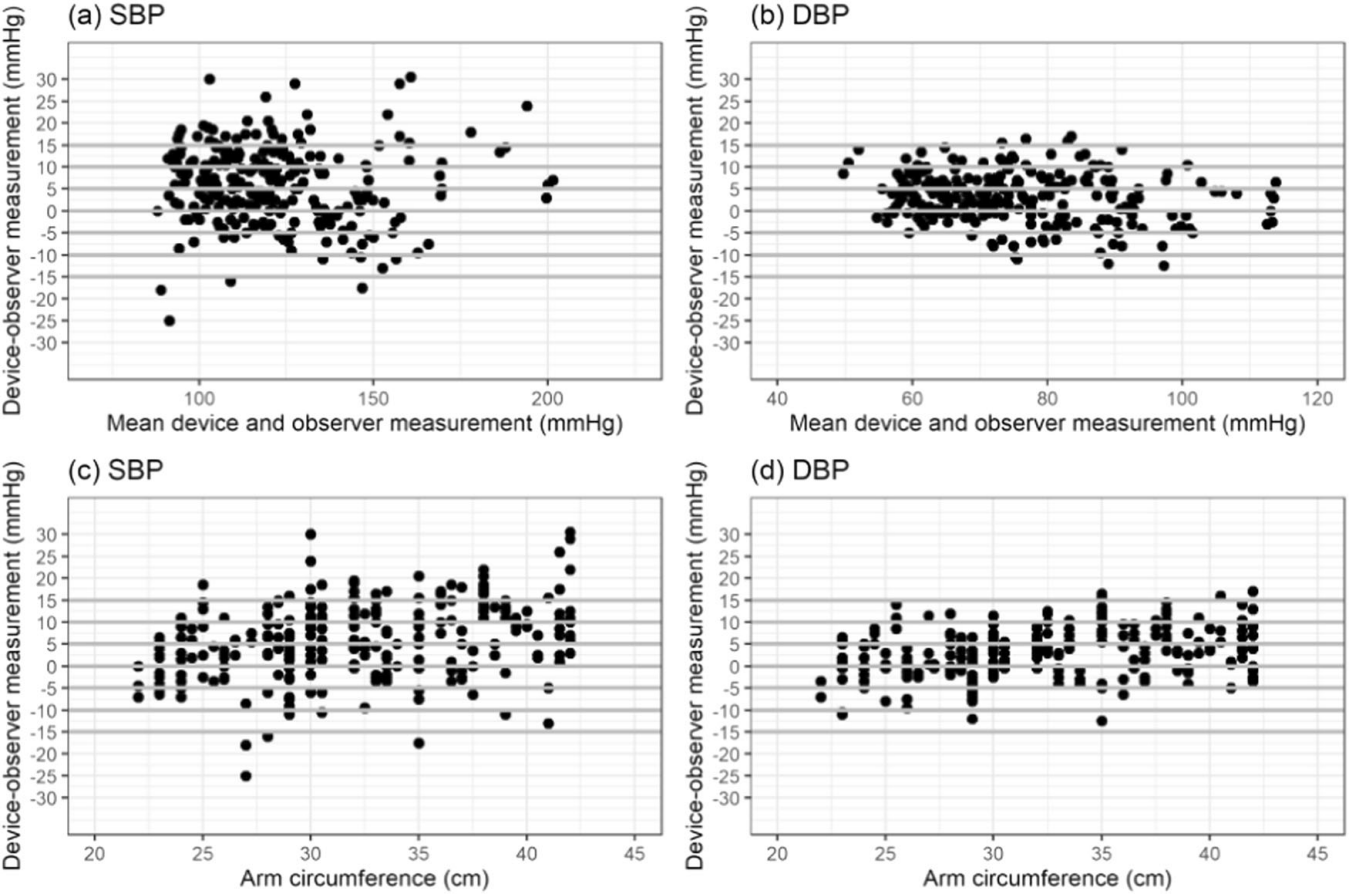
Bland-Altman Plots for BP3MW1-4YCVS. Bland–Altman Plots of the differences between the BP3MW1-4YCVS measurements and observer measurements for (**a**) systolic blood pressure (SBP) and (**b**) diastolic blood pressure (DBP) and for (**c**) SBP by arm circumference and (**d**) DBP by arm circumference.

Bland–Altman scatter plots of the differences between the device under test and observer measurements for SBP and DBP against their average value and scatterplots of test-reference BP differences by subject’s arm circumference are shown in Figs. 1–5.

## Discussion

We tested five automated blood pressure monitors intended for home use using the ISO 81060-2:2018/AMD 1:2020 standard. Two devices (BP5450 and BP7450) fulfilled this standard for a general adult population, although for BP5450 the standard deviation of the subject-level diastolic blood pressure difference was borderline. UA-8000WM, WGNBPA-240BT and BP3MW1-4YCVS did not meet this validation standard in our test. Using the ANSI/AAMI/ISO 81060-2:2018 + A1:2020 protocol as a guide, we removed measurements with discrepant observer readings based on our interpretation of the technical document and the recommendations we received from an expert consultant who trained the study team.

These findings suggest that the accuracy of automated home blood pressure monitors available in the U.S. market that have not been validated cannot be assumed and underscores the importance of verifying the validation status of at-home blood pressure monitors prior to use for diagnosis or clinical monitoring. Prior observers have noted that many BP monitors available for home use have not been validated [10, 11], and that accuracy is lower in non-validated devices than validated devices [7, 12, 13].

Since monitors that have been validated for accuracy are recommended for BP measurement, it is important that consumers and patients are advised to check whether a blood pressure monitor has been validated for accuracy [14]. Fortunately, there are readily available websites consumers can use to determine the validation status of home blood pressure monitors [15, 16].

### Strengths and limitations

Strengths of this study include the testing of multiple commercial home BP monitors under similar rigorous conditions and achieving the required distribution of subjects by arm size, blood pressure and sex. Limitations are that the study population was limited to a general adult population, and the generalizability of these findings to other populations (e.g., pregnant women, children) is unknown. Additionally, while we followed the recommendations for which data points to include or exclude from an expert consultant who trained the study team, we removed and repeated reference measurements with discrepant observer readings but did not remove and repeat the preceding test device measurement when the repeated reference measurement met the criteria for observer agreement. While we believe that this choice is consistent with the ISO 81060-2:2018 + A1:2020 protocol [8, 9], we acknowledge that this interpretation differs from the one described by Stergiou et al. [17].

### Summary

#### What is known about this topic

- The importance of out-of-office BP is increasingly recognized for hypertension diagnosis and management.
- Many devices marketed for home use do not have published validation information available testing them using an international standard.

#### What this study adds

- BP5450 and BP7450 are accurate enough for home use in a general adult population.
- UA-8000WM, WGNBPA-240BT and BP3MW1-4YCVS did not meet the applied ISO standard for home use.

## Supplementary information

Supplement Table 1

## Data Availability

Final study data is available by written request to the investigators.
