# Supplementary material for: Validation testing of five home blood pressure monitoring devices for the upper arm according to the ISO 81060-2:2018/AMD 1:2020 protocol
Source: J Hum Hypertens. 2023 Jan 18;37(2):134–40. doi: 10.1038/s41371-022-00795-6 (PMC9957726; doi:10.1038/s41371-022-00795-6)
Supplement: Supplementary file 1 — Supplement Table 1 [file 41371_2022_795_MOESM1_ESM.docx]

**Supplementary Table 1:** Data in supplementary table 1 represents arm circumference count and percentage in each monitor’s arm circumference distribution.

| **BP7450** | Count | % |
| --- | --- | --- |
| 22.0 - 24.5 cm, lowest octile | 9 | 10.6 |
| 22.0 - 27.0 cm, first quartile | 18 | 21.2 |
| 22.0 - 32.0 cm, second quartile | 48 | 56.5 |
| 32.0 - 42.0 cm, third quartile | 37 | 43.5 |
| 37.0 - 42.0 cm, fourth quartile | 17 | 20.0 |
| 39.5 - 42.0 cm, highest octile | 9 | 10.6 |
| **BP5450** |  |  |
| 17.0 - 20.0 cm, lowest octile | 9 | 10.6 |
| 17.0 - 23.2 cm, first quartile | 17 | 20.0 |
| 17.0 - 29.5 cm, second quartile | 34 | 40.0 |
| 29.6 - 42.0 cm, third quartile | 52 | 61.2 |
| 35.9 - 42.0 cm, fourth quartile | 22 | 25.9 |
| 39.0 - 42.0 cm, highest octile | 12 | 14.1 |
| **UA-8000WM** |  |  |
| 23.0 - 25.5 cm , lowest octile | 9 | 10.6 |
| 23.0 - 28.0 cm , first quartile | 17 | 20.0 |
| 23.0 - 33.0 cm , second quartile | 47 | 55.3 |
| 33.0 - 43.0 cm , third quartile | 38 | 44.7 |
| 38.0 – 43.0 cm, fourth quartile | 20 | 23.5 |
| 40.5 – 43.0 cm, highest octile | 9 | 10.6 |
| **WGNBPA-240BT** |  |  |
| 23.0 - 25.5 cm , lowest octile | 10 | 11.8 |
| 23.0 - 28.0 cm , first quartile | 17 | 20.0 |
| 23.0 - 33.0 cm , second quartile | 48 | 56.5 |
| 33.0 - 43.0 cm , third quartile | 37 | 43.5 |
| 38.0 – 43.0 cm, fourth quartile | 17 | 20.0 |
| 40.5 – 43.0 cm, highest octile | 10 | 11.8 |
| **BP3MW1-4YCVS** |  |  |
| 22.0 - 25.5 cm, lowest octile | 9 | 10.6 |
| 23.0 - 28.0 cm , first quartile | 17 | 20.0 |
| 23.0 - 33.0 cm, second quartile | 45 | 52.9 |
| 33.0 - 43.0 cm , third quartile | 40 | 47.1 |
| 38.0 – 43.0 cm, fourth quartile | 21 | 24.7 |
| 40.5 – 42.0 cm, highest octile | 12 | 14.1 |
